# Evaluation by simulation of clinical trial designs for evaluation of treatment during a viral haemorrhagic fever outbreak

**DOI:** 10.1186/s12874-021-01287-w

**Published:** 2021-05-06

**Authors:** Pauline Manchon, Drifa Belhadi, France Mentré, Cédric Laouénan

**Affiliations:** 1 INSERM, Centre d’Investigation clinique-Epidémiologie Clinique 1425, Hôpital Bichat, F-75018 Paris, France; 2 Département Epidémiologie Biostatistiques et Recherche Clinique, AP-HP, Hôpital Bichat, F-75018 Paris, France; 3 Université de Paris, INSERM, IAME UMR 1137, F-75018 Paris, France

**Keywords:** Viral haemorrhagic fever outbreak, Clinical trial design, Simulation study

## Abstract

**Background:**

Viral haemorrhagic fevers are characterized by irregular outbreaks with high mortality rate. Difficulties arise when implementing therapeutic trials in this context. The outbreak duration is hard to predict and can be short compared to delays of trial launch and number of subject needed (NSN) recruitment. Our objectives were to compare, using clinical trial simulation, different trial designs for experimental treatment evaluation in various outbreak scenarios.

**Methods:**

Four type of designs were compared: fixed or group-sequential, each being single- or two-arm. The primary outcome was 14-day survival rate. For single-arm designs, results were compared to a pre-trial historical survival rate p_H_. Treatments efficacy was evaluated by one-sided tests of proportion (fixed designs) and Whitehead triangular tests (group-sequential designs) with type-I-error = 0.025. Both survival rates in the control arm p_C_ and survival rate differences Δ (including 0) varied. Three specific cases were considered: “standard” (fixed p_C_, reaching NSN for fixed designs and maximum sample size N_Max_ for group-sequential designs); “changing with time” (increased p_C_ over time); “stopping of recruitment” (epidemic ends). We calculated the proportion of simulated trials showing treatment efficacy, with K = 93,639 simulated trials to get a type-I-error PI_95%_ of [0.024;0.026].

**Results:**

Under H_0_ (Δ = 0), for the “standard” case, the type-I-error was maintained regardless of trial designs. For “changing with time” case, when p_C_ > p_H,_ type-I-error was inflated, and when p_C_ < p_H_ it decreased. Wrong conclusions were more often observed for single-arm designs due to an increase of Δ over time. Under H_1_ (Δ = + 0.2), for the “standard” case, the power was similar between single- and two-arm designs when p_C_ = p_H_. For “stopping of recruitment” case, single-arm performed better than two-arm designs, and fixed designs reported higher power than group-sequential designs. A web R-Shiny application was developed.

**Conclusions:**

At an outbreak beginning, group-sequential two-arm trials should be preferred, as the infected cases number increases allowing to conduct a strong randomized control trial. Group-sequential designs allow early termination of trials in cases of harmful experimental treatment. After the epidemic peak, fixed single-arm design should be preferred, as the cases number decreases but this assumes a high level of confidence on the pre-trial historical survival rate.

**Supplementary Information:**

The online version contains supplementary material available at 10.1186/s12874-021-01287-w.

## Background

Emerging infectious diseases such as influenza, cholera, coronavirus, varicella, meningitis and viral haemorrhagic fevers recently caused numerous outbreaks [1]. Since 2003, the coronavirus caused various outbreaks around the world. SARS-CoV-1 was first observed in Southeast Asia. In 2012, MERS-CoV caused a case-fatality rate of 37% in the Middle East [2]. Today SARS-CoV-2 is pandemic. Viral haemorrhagic fevers are among the most severe, including Crimean–Congo haemorrhagic fever, Ebola virus disease, Lassa fever and Marburg haemorrhagic fever, which are endemic in some areas of Africa, South America and Asia [3]. They are characterized by outbreaks with high mortality rate which occur irregularly and are hard to anticipate. The second largest outbreak of Ebola virus disease since the 2014–2016 one in West Africa, began in 2018 and ended in June 2020 in the Democratic Republic of the Congo [4].

Recently, a scoping review evaluated political, economic, administrative, regulatory, logistical, ethical and social (PEARLES) challenges associated with clinical research in the context of emergency epidemics [5]. The authors highlighted the challenges associated with the planning, conduct and dissemination of clinical research responses during an epidemic. They stressed the need for developing solutions to improve rapid clinical research deployment, delivery, and dissemination for future epidemics.

Difficulties often occur when implementing therapeutic trials in the context of viral haemorrhagic fever outbreak. Indeed, the duration of the outbreak is hard to predict and can be very short compared to the necessary delay to launch a trial and recruit the required number of patients. A reduced number of included patients impact the statistical power of the trial and can lead to study which cannot demonstrate a significant effect of their experimental treatment [6–8]. Evolution of the number of cases and case fatality rate during an outbreak period is also a dimension to be considered when designing a clinical trial in this context.

Moreover, the design of the study must be acceptable on the field by patients, healthcare workers and the global population. In the situation of an epidemic peak, local authorities and non-governmental organizations managing treatment centres may argue that randomization is hard to implement due to the high case-fatality rate and the reluctance to give a less beneficial treatment to part of the patients [7, 9, 10]. A single-arm design may seem to be best suited for those ethical aspects, its feasibility on the field and its acceptability by the population affected directly or indirectly by the disease. Nevertheless, non-comparative trials can lead to substantially biased results if the pre-trial survival rate is incorrect, indeed its evolution over time is not taken into account.

Another aspect to consider is the choice of the design: fixed or group-sequential. Indeed, group-sequential designs sometimes lead to reducing the duration of the trial especially in case of early stop for futility or efficacy of the experimental treatment [11]. At each interim analysis, the efficacy of the treatment is tested to decide if the recruitment has to continue or if the experimental treatment is already shown to be effective or not. Group-sequential designs are interesting in an epidemic peak of infectious disease, under emergency conditions and with different treatment candidates.

During the Ebola outbreak in West Africa, between December 2013 and May 2016, three antivirals (favipiravir, brincidofovir, TKM-13083), one cocktail of antibodies, and convalescent plasma were evaluated in five therapeutic trials in Guinea, Liberia and Sierra Leone [6–8, 12, 13]. These trials were implemented during the second phase of the outbreak, when the number of cases decreased, and none of them achieved the number of patients required. For four trials, the primary outcome was the mortality at 14 days after the inclusion. This outcome was chosen because it requires no invasive procedures or special equipment, and most of the time deaths from Ebola occur within 14 days. Only one used the mortality at 28 days and it was the only randomized trial with an adaptive scheme [6]. The four other trials were non-randomized and the comparison was established with the pre-trial mortality based on historical data, and three trials used a group-sequential design [7, 12, 13]. More recently, a multi-arm multi-stage clinical trial was conducted with four investigational therapies for Ebola virus disease in the Democratic Republic of Congo, where, two of the four treatments compared were stopped based on interim analysis [14].

A simulation study by Cooper et al. [15] was conducted to investigate the choice of the study design in the context of the Ebola Virus disease. In particular, they worked on a multi-stage approach (MSA) comprising a single-arm phase II study followed by one or two phase III studies. They concluded that the MSA and group-sequential double-arm randomised trial led to substantially fewer deaths than a conventional two-arm randomised trial if the tested interventions were either highly effective or harmful. MSA was applied to design two clinical trials during the 2014–2015 West African Ebola outbreak [16].

The objectives of the present clinical trial simulation study was to compare different designs, during various outbreak scenarios to develop recommendations when designing a clinical trial for viral haemorrhagic fever. We considered several specific cases associated with the timeline of an outbreak. Indeed, at the beginning of an outbreak, standard cares are developed, which lead to increased survival rates. After the epidemic peak, it is more difficult to include patients in a trial due to a decreased number of cases. This crisis situation requires an accelerated research process and the study feasibility depends on the choice of the trial design. For this simulation study, we choose to compare four clinical trial designs: fixed single-arm, group-sequential single-arm, fixed two-arm and group-sequential two-arm designs. An online tool based on the results from this simulation study was also developed.

## Methods

We performed clinical trial simulations to compare four designs (see Additional file 1): fixed single-arm trial (F1); group-sequential single-arm trial (S1); fixed two-arm trial (F2); and group-sequential two-arm trial (S2). A randomization 1:1 was considered for two-arm designs. The primary outcome of each simulated trial was the number of patients who survived at day 14 after inclusion. For single-arm designs, results were compared to a pre-trial historical survival rate.

Several outbreak scenarios were explored based on three scenario for the timeline of the outbreak and various values of control survival rate (p_C_) and efficacy of the treatment (Δ). A scenario was defined as the combination of one specific case of outbreak timeline, one control survival rate and one survival rate difference.

### Three specific cases of outbreak timeline (Fig. 1)

The “standard” case corresponded to a trial launch at the beginning of the outbreak, i.e. a fixed p_C_ and a number of subjects equal to the number of subjects needed (NSN) for fixed designs and the possibility to reach the maximum sample size (N_Max_), if needed for group-sequential trials.

**Fig. 1:**
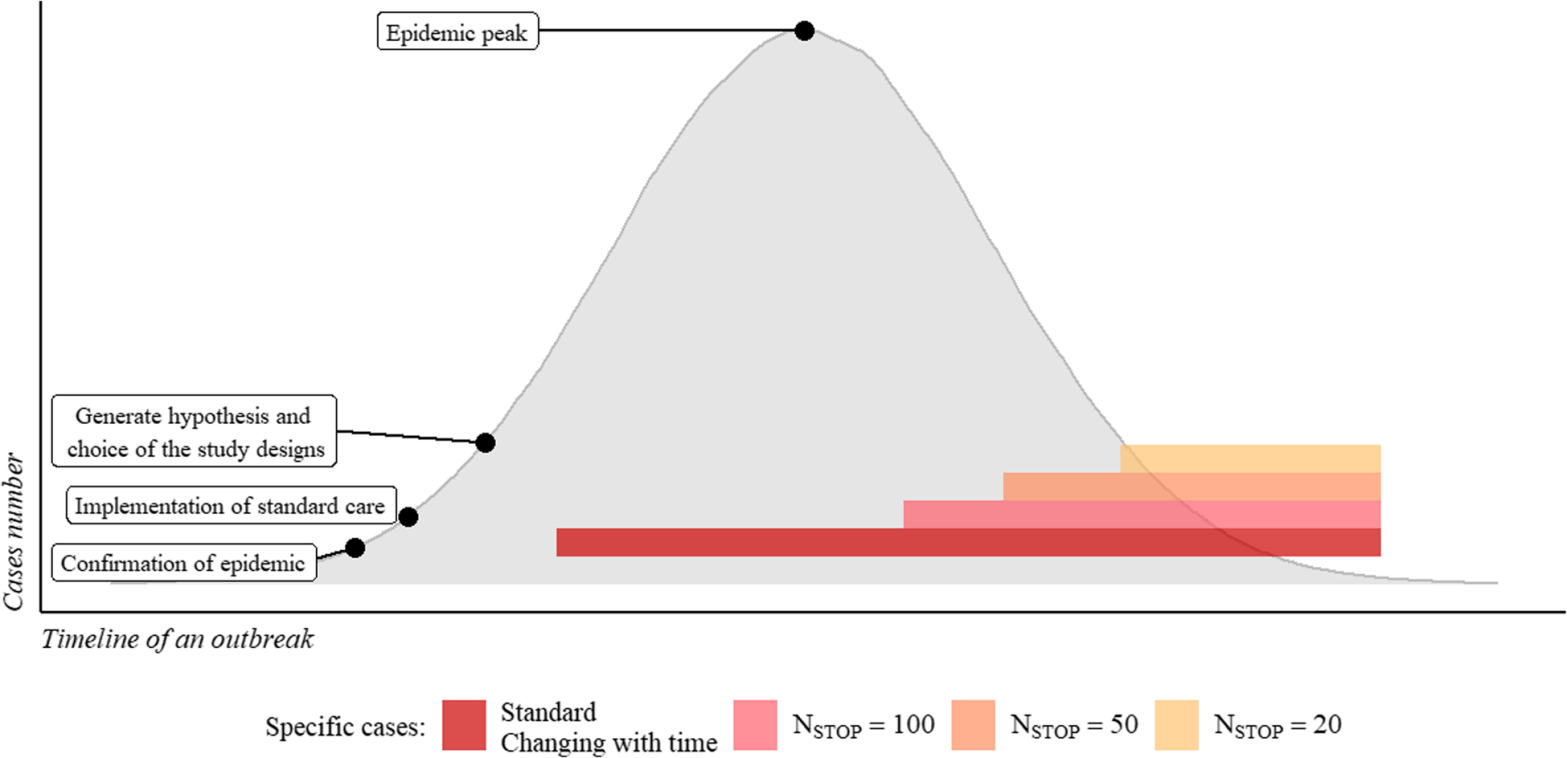
Outbreak timeline and specific cases simulated. Three specific cases of outbreak timeline were simulated: “standard” specific case with a fixed control survival rate; “Changing with time” specific case with an increase of the control survival rate over time; and “stopping of recruitment” specific case with a fixed control survival rate and an early stop of the trial due to an outbreak end. Several sample sizes were simulated for the “stopping of recruitment” case: N_STOP_ = 100, N_STOP_ = 50, and N_STOP_ = 20

The “changing with time” case was defined to mimic increased proportion of patients who survived in the control group over time during the outbreak (p_C_). An increase of + 0.03 every 20 patients included was simulated, with a maximal total increase of 0.10. This scenario was extrapolated from the real weekly evolution of the survival rate during the 2014’s Ebola epidemic in West Africa [17].

The “stopping of recruitment” case was performed to mimic the effect of starting recruitment at different times during the time course of the outbreak after the epidemic peak. Several sample sizes were simulated: N_STOP_ = 20, N_STOP_ = 50, and N_STOP_ = 100, and we assumed a fixed p_C_ not changing with time. If required, the *p*-values in group-sequential designs were adjusted for underrunning (group-sequential trial which ended before that the stopping rule has been fulfilled) [18].

### Statistical tests

For fixed designs (F1 and F2), the treatment efficacy was evaluated by a one-sided comparison test of proportions. A Yate’s continuity correction was applied if needed. The experimental treatment was considered significantly superior if the *p*-value was inferior to 0.025 with the power 1-β = 0.90. For group-sequential designs (S1 and S2), Whitehead triangular one-sided tests were conducted [19]. Stopping boundaries to conclude to the treatment efficacy were calculated using a type-I-error α = 0.025 and a power 1-β = 0.90. Group-sequential analyses were conducted every 20 patients included for both single and two-arm trials; and at each interim analysis the statistics Z (cumulative benefit of experimental treatment) and V (global information) from the Whitehead triangular test were calculated. The stopping rules for an interim analysis *j* were then defined as follows:

- ▪ If ${\mathrm{Z}}_{\mathrm{j}}\ge \mathrm{a}+\mathrm{c}{\mathrm{V}}_{\mathrm{J}}-0.583\sqrt{{\mathrm{V}}_{\mathrm{j}}-{\mathrm{V}}_{\mathrm{j}-1}}$, the trial was stopped as the experimental treatment was shown to be significantly better than the control;
- ▪ If ${\mathrm{Z}}_{\mathrm{j}}\le -\mathrm{a}+3\mathrm{c}{\mathrm{V}}_{\mathrm{j}}+0.583\sqrt{{\mathrm{V}}_{\mathrm{j}}-{\mathrm{V}}_{\mathrm{j}-1}}$, the trial was stopped due to futility/inefficacy of the experimental treatment;
- ▪ Otherwise, the trial continued.

The stopping rules for the final analysis J were defined by:

- ▪ If ${\mathrm{Z}}_{\mathrm{J}}\ge \mathrm{a}+\mathrm{c}{\mathrm{V}}_{\mathrm{J}}-0.583\sqrt{{\mathrm{V}}_{\mathrm{J}}-{\mathrm{V}}_{\mathrm{J}-1}}$, the experimental treatment was considered as significantly better than the control;
- ▪ Otherwise, efficacy was not shown.

In case of premature termination of the group-sequential trial:

- ▪ If premature stopping of the trial occurred due to external reasons, i.e. independently of the cumulative information and the observed difference in efficacy between treatments, the final *p*-values were adjusted by the method proposed by Whitehead for underrunning [18],
- ▪ Otherwise, efficacy was not shown.

### Sample sizes

The basic scenario was used to calculate the sample sizes. It assumed a “standard” case for the outbreak, a proportion of patient who survived at day 14 p_C_ = 0.5 (control survival rate) for two-arm trials, and p_H_ = 0.5 (pre-trial historical survival rate) for single-arm trials, and an expected survival rate difference Δ =  + 0.2. The number of subjects needed (NSN) for each fixed design and the maximum sample size for each group-sequential design (N_Max_), with a power 1-β = 0.9 and a one-sided type-I-error α = 0.025, were calculated and were: NSN_F1_ = 60, NSN_F2_ = 248, N_MAX, S1_ = 91, N_MAX, S2_ = 378.

For group-sequential trials, when the final analysis was performed with a cumulated number of subjects included equal to N_Max_, the final analysis J was conducted with the remaining patients, necessarily inferior to 20 patients (number of patients included at each intermediate analysis).

Those sample sizes were used for all simulated scenario under “standard” or “changing with time” cases.

### Simulated values of parameters

All specific cases of outbreak timeline and all designs were simulated with different values of the survival rate in the control arm p_C_ = (0.35; 0.50; 0.75) and survival rate difference Δ = (−0.10; 0; 0.10; 0.20). Of note Δ = 0 corresponds to simulation under the null hypothesis. The experimental survival rate in the treatment arm was defined as p_E_ = p_C_ + Δ. When p_C_ + Δ ≤ 0 then p_E_ was set at 0.01 in order to be able to calculate the log-odds ratio for triangular tests. Likewise, p_E_ was set at 1 when p_C_ + Δ > 1.

For single-arm trials the fixed pre-trial historical survival rate p_H_ was assumed to be 0.50 regardless of the scenario and control survival rate.

### Design evaluation

For each design and each scenario, defined by the specific case of outbreak timeline and parameters p_C_ and Δ, K = 93,639 simulated trials were simulated in order to ensure a 95% prediction interval width of 0.001 around α = 0.025. The seed was chosen randomly and was the same for all the scenarios. We set the seed once at the beginning of the entire set of simulated studies.

To evaluate the performance of each design, for each scenario, we calculated the proportion of simulated trials showing the efficacy of the experimental treatment i.e. the proportion of significant tests (denoted p), which corresponds to the type-I-error when Δ = 0, and the median [with 5th and 95th percentiles] number of subjects included in group-sequential trials (to be compared with the NSN of the fixed trials).

Simulations were performed with R software version 3.2.1.

## Results

### Scenarios under H_0_ (∆ = 0)

For the basic scenario (p_C_ = 0.50 and ∆ = 0), the type-I-error was maintained regardless of the trial design (Table 1). For the “changing with time” case, the type-I-error was calculated for one-arm designs exclusively: for two-arm designs, control and experimental survival rates increased together. Moreover, the type-I-error for single-arm designs F1 and S1 increased respectively to *p* = 0.069 and *p* = 0.062. This was due to the incorrect assumption on the pre-trial historical survival rate set at p_H_ = 0.50 whereas the survival rate increased during the trial conduct, leading to a p_H_ inferior to the control survival rate p_C_. The assumption on the pre-trial historical survival rate is very important. Indeed, a wrong assumption on the value of the pre-trial historical survival rate (p_H_) has considerable impact on the results. For the “standard” and “changing with time” cases, when p_C_ = 0.75, whereas we assumed p_H_ = 0.50, *i.e*, p_H_ < p_C_, and ∆ = 0, single-arm trials (either fixed or group-sequential) still had higher proportions of simulated trials showing a significant efficacy than two-arm trials. On the contrary, when p_H_ > p_C_, with p_C_ = 0.35 and ∆ = 0, proportions of simulated trials showing a significant efficacy was very close to zero. Of note, for the “changing with time” case, when p_C_ = 0.75 and ∆ = 0, the type-I-error was lower for group-sequential two-arm design (*p* = 0.015) than fixed two-arm design (*p* = 0.025). This was due to the presence of 11% of inconclusive trials for the group-sequential design (data not shown).

**Table 1:** Proportion of trials showing significant improvement with the experimental treatment (“standard” and “changing with time” cases)

	Design	p_C_ = 0.50				p_C_ = 0.35		p_C_ = 0.75	
		∆ = 0.20	∆ = 0.10	∆ = 0	∆ = − 0.10	∆ = 0.20	∆ = 0	∆ = 0	∆ = − 0.10
Standard	F1	0.896	0.351	**0.025**	0.0002	0.120	0.000	0.985	0.662
	S1	0.893	0.339	**0.024**	0.0003	0.115	0.0001	0.982	0.657
	F2	0.897	0.347	**0.024**	0.0001	0.887	**0.025**	**0.025**	0.0001
	S2	0.904	0.339	**0.025**	0.0004	0.883	**0.025**	**0.024**	0.0002
Changing with time	F1	0.964	0.536	0.069	0.0011	0.241	0.0001	0.997	0.823
	S1 (^a^)	0.944 (0)	0.520 (0)	0.062 (0)	0.0008 (0)	0.229 (0)	0 (0)	0.991 (0)	0.796 (0)
	F2	0.926	0.370	**0.025**	0.0002	0.875	0.023	**0.025**	0.0001
	S2 (^a^)	0.958 (0.0007)	0.390 (0.0001)	**0.024** (0)	0.0004 (0)	0.880 (0)	**0.025 (0)**	0.015 (0.11)	0 (0)

In bold, type-I-errors maintained in [0.024;0.026]

*Abbreviations*: p_C_ indicates control survival rate; ∆: simulated survival rate difference; F1: fixed single-arm design; S1: group-sequential single-arm design; F2: fixed two-arm design; S2: group-sequential two-arm design; Standard: specific case with a fixed control survival rate; Changing with time: specific case with an increase of the control survival rate over time

(^a^): Proportion of significant tests with proportion of inconclusive group-sequential trials for “changing with time” case

Regarding the “stopping of recruitment” case, the only scenario where the type-I-error was maintained was observed under H_0_ for the group-sequential single-arm design with p_C_ = 0.50, ∆ = 0 and N_STOP_ = 100, a sample size very close to the N_max,S1_ (Table 2). When inclusions were poor and p_C_ = 0.50, ∆ = 0, the type-I-error was always lower. For this specific case with p_C_ = 0.75, ∆ = 0 and N_STOP_ = 50, wrong assumption on p_H_ had a stronger impact on the type-I-error estimated at 0.95 to 0.97 for single-arm designs.

**Table 2:** Proportion of trials showing significant improvement for “stopping of recruitment” cases

Stopping of recruitment	Design		p_C_ = 0.50			p_C_ = 0.75		
			∆ = 0.20	∆ = 0	∆ = − 0.10	∆ = 0.20	∆ = 0	∆ = − 0.10
N_STOP_ = 20	F1		0.416	0.021	0.002	1	0.618	0.244
	S1	(^a^)	0.414 (0.75)	0.021 (0.587)	0.002 (0.245)	1 (0.003)	0.615 (0.585)	0.242 (0.830)
		(^b^)	0.234 + 0.180	0.006 + 0.015	0.0004 + 0.0012	0.997 + 0.002	0.411 + 0.204	0.116 + 0.126
	F2		0.054	0.006	0.002	0.048	0.005	0.001
	S2	(^a^)	0.118 (1)	0.042 (1)	0.060 (1)	0.054 (1)	0.019 (1)	0.044 (1)
		(^b^)	0 + 0.118	0 + 0.042	0 + 0.060	0 + 0.054	0 + 0.019	0 + 0.044
N_STOP_ = 50	F1		0.858	0.032	0.0005	1	0.972	0.619
	S1	(^a^)	0.793 (0.136)	0.023 (0.020)	0.0004 (0.0002)	1 (0)	0.948 (0.054)	0.526 (0.181)
		(^b^)	0.720 + 0.073	0.016 + 0.007	0.0003 + 0.0001	1 + 0	0.916 + 0.032	0.438 + 0.088
	F2		0.333	0.032	0.005	0.320	0.021	0.003
	S2	(^a^)	0.234 (0.987)	0.016 (0.950)	0.003 (0.837)	0.420 (0.998)	0.020 (0.989)	0.017 (0.903)
		(^b^)	0.013 + 0.221	0.0001 + 0.016	0 + 0.003	0.002 + 0.418	0 + 0.020	0 + 0;017
N_STOP_ = 100	F1		0.988	0.028	0	1	1	0.875
	S1		0.894 (0)	**0.025** (0)	0.0005 (0)	1 (0)	0.981 (0)	0.661 (0)
	F2		0.542	0.029	0.002	0.834	**0.024**	0.0008
	S2	(^a^)	0.425 (0.732)	0.012 (0.529)	0.0004 (0.190)	0.747 (0.765)	0.012 (0.766)	0.0004 (0.287)
		(^b^)	0.255 + 0.170	0.005 + 0.007	0.0001 + 0.0003	0.236 + 0.511	0.001 + 0.011	0 + 0.0004

In bold, type-I-errors maintained in [0.024;0.026]

*Abbreviations*: p_C_ indicates control survival rate; ∆: simulated survival rate difference; F1: fixed single-arm design; S1: group-sequential single-arm design; F2: fixed two-arm design; S2: group-sequential two-arm design; Stopping of recruitment: specific case with a fixed control survival rate and an early stop of the trial due to an outbreak end; For group-sequential trials, the total of trials demonstrating an efficacy of the experimental treatment was defined by the sum of trials with a significant test and trials with an adjusted *p*-value inferior to 0.025. The *p*-value was adjusted for underrunning using the method proposed by Whitehead [18]

(^a^): Proportion of significant tests with proportion of inconclusive group-sequential trials for “changing with time” cases

(^b^): Proportion of significant tests + proportion of trials with adjusted *p*-values inferior to 0.025 for group-sequential trials and “stopping of recruitment” cases

### Scenarios under H_1_ (∆ = 0.2)

For the “standard” case, proportions of simulated trials with a significant efficacy were similar (*p* = 0.90) between single- and two-arm designs under H_1_ when the control survival rate was equal to the pre-trial historical survival rate (p_C_ = p_H_ = 0.50) and ∆ = 0.2 (Table 1). However, when the pre-trial historical survival rate was incorrect, i.e. the control survival rate was lower (p_C_ = 0.35) than the pre-trial historical survival rate, power of single-arm designs was poor. Indeed, only 11–12% of simulated single-arm trials were significant when p_C_ = 0.35 and p_E_ = 0.55 (∆ = 0.20), versus 88–89% of the simulated two-arm trials. For “changing with time” cases, proportions were overall similar between fixed and group-sequential designs (Table 1). Regarding “stopping of recruitment” cases, single-arm trials performed better than two-arm trials (Table 2). When p_C_ = 0.50, the proportions of simulated trials showing a significant efficacy were higher for single-arm trials, ranging from 41% (N_STOP_ = 20) to 99% (N_STOP_ = 100), compared to two-arm trials (from 5% for N_STOP_ = 20 to 54% for N_STOP_ = 100). Moreover, fixed designs reported higher significant efficacy than group-sequential designs when ∆ = 0.20.

### Scenarios with ∆ = − 0.1

When the treatment was harmful (∆ = − 0.1), the proportion of simulated trials showing the efficacy of the experimental treatment was close to 0 regardless of the specific case, except when p_C_ = 0.75 for single-arm designs, which was due to the wrong assumption on p_H_ (Tables 1 and 2).

### Number of subjects included

**Fig. 2:**
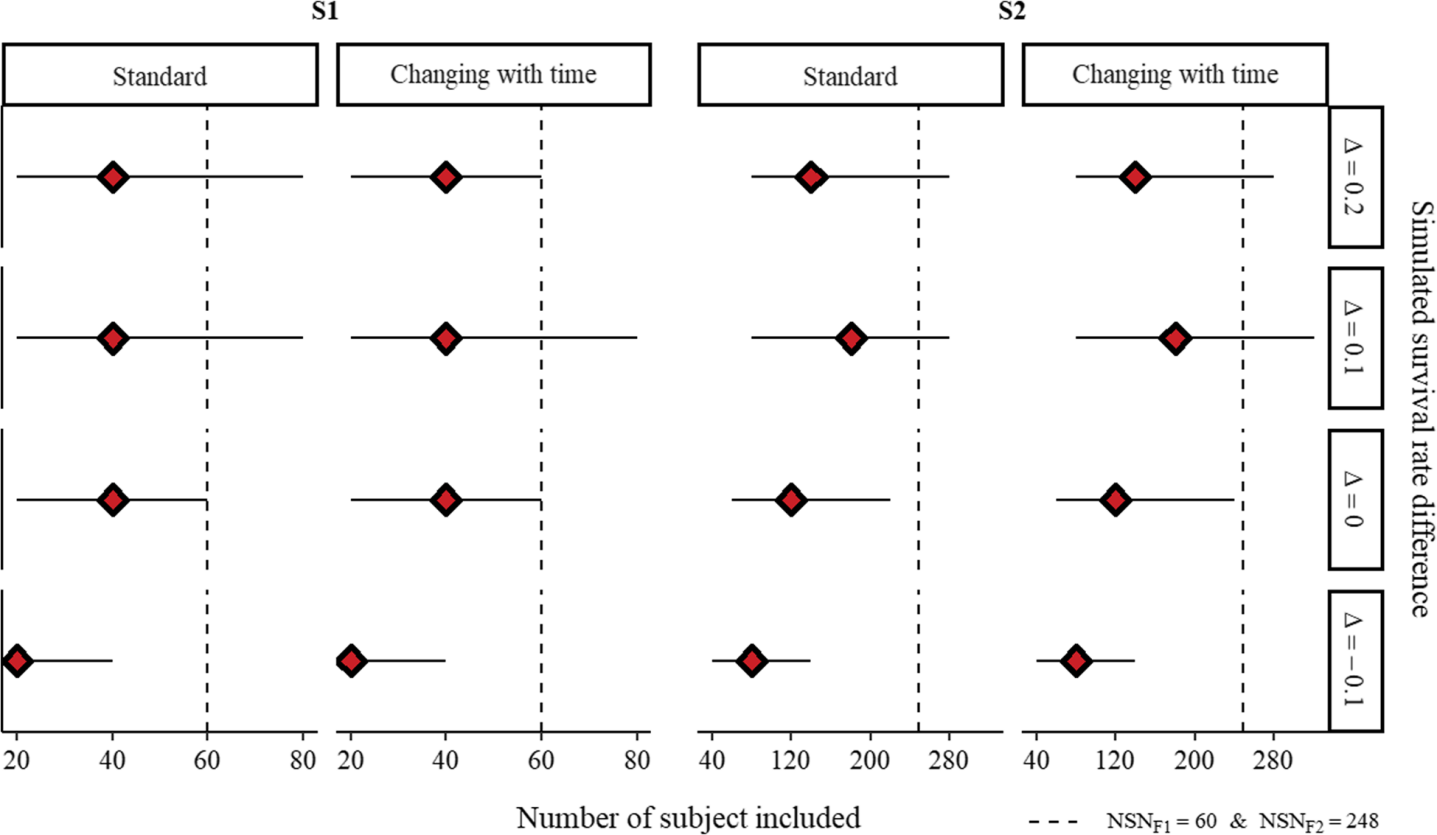
Number of subjects included in sequential trials “standard” and “changing with time” specific cases, (p_C_ = 0.50). Red diamonds denoted median of subject included and lines denoted 5th and 95th percentiles. Dashed lines corresponded to NSN_F1_ = 60 and NSN_F2_ = 248. Abbreviations: p_C_ indicates control survival rate; ∆: simulated survival rate difference; F1: fixed single-arm design; S1: group-sequential single-arm design; F2: fixed two-arm design; S2: group-sequential two-arm design; “Standard”: specific case with a fixed control survival rate; “Changing with time”: specific case with an increase of the control survival rate over time

The median number [P5^th^-P95^th^] of subject included in group-sequential trials for both “standard” and “changing with time” cases are reported in Fig. 2 with p_C_ = 0.50. The median number of included subjects was always lower in the group-sequential trials compared with the fixed trials, especially when the experimental treatment effect was harmful (∆ =  − 0.10). Indeed, scenarios with ∆ =  − 0.10 or ∆ = 0 always presented a median number of included subjects lower than scenarios with ∆ > 0. Moreover, less than 10% of the group-sequential single- or two-arm trials reported a higher number of included subjects than single- or two-arm fixed trials respectively (Table 3). On the other hand, the NSN and N_Max_ determined for two-arm designs were greater to 200 patients whereas the NSN and N_Max_ for single-arm designs were lower to 100 patients. The rapidity to perform a single-arm trial compared with two-arm designs, which require more than the double of inclusions, is considerable.

**Table 3:** Proportion of group-sequential trials with subjects numbers superior to fixed designs NSN (p_C_ = 0.50)

		∆ = 0.2	∆ = 0.1	∆ = 0	∆ = − 0.1
Standard	S1	0.07	0.13	0.02	0.0004
	S2	0.09	0.15	0.02	0.0003
Changing with time	S1	0.02	0.13	0.04	0.001
	S2	0.1	0.23	0.03	0.0005

*Abbreviations*: p_C_ indicates control survival rate; ∆: simulated survival rate difference; F1: fixed single-arm design; S1: group-sequential single-arm design; F2: fixed two-arm design; S2: group-sequential two-arm design; “Standard”: specific case with a fixed control survival rate; “Changing with time”: specific case with an increase of the control survival rate over time

### Online tool to visualize results of simulated scenarios

Simulation results were reported online on the following dashboard: https://pmn-bch.shinyapps.io/simu-vhf/. This tool reports all scenarios for all designs (F1, F2, S1, S2), according to the choice of parameters: the control survival rate p_C_ and the survival rate difference ∆. Available values are, for p_C_: 0.35, 0.40, 0.45, 0.50, 0.65, 0.75, and for ∆:-0.40, − 0.20, − 0.10, 0, 0.10, 0.20, 0.30, 0.40. Several specific cases of an outbreak can be selected: the “standard” one (with fixed p_C_, the NSN for fixed designs and N_Max_ if required for group-sequential trials), the “changing with time” one which simulated the evolution of the survival rate and the “stopping of recruitment” which simulated an early stop of the trial (with N_STOP_ = 20; N_STOP_ = 50; N_STOP_ = 100). This tool will allow to evaluate all the elements before designing a clinical trial in the context of an outbreak of a viral haemorrhagic fever.

## Discussion

We presented results from our simulation to help future investigators choosing the best clinical trial design (single- versus two-arm, fixed versus group-sequential trial) based on the outbreak timeline of an emerging infectious disease.

At the beginning of an outbreak group-sequential two-arm trials should be preferred. A group-sequential design will often allow an early termination of the trial when the treatment does not perform better than the control or when treatment efficacy is large. Moreover, the number of infected cases increases until the epidemic peak. Thus, the required number of patients to perform a two-arm trial can be reached and the potential evolution of the survival rate will be considered with the presence of the control arm.

For trials beginning after the epidemic peak fixed single-arm design should be performed. As the number of cases decreases after the peak of the epidemic, single-arm designs would be preferred as they required a lower number of patients than two-arm designs. However, this assumes that the pre-trial historical survival rate was correctly estimated. We showed that with an incorrect assumption on this survival rate, the type-I-error is not maintained. Single-arm trials should only be conducted if a high level of confidence can be put on the pre-trial historical survival rate used.

**Fig. 3:**
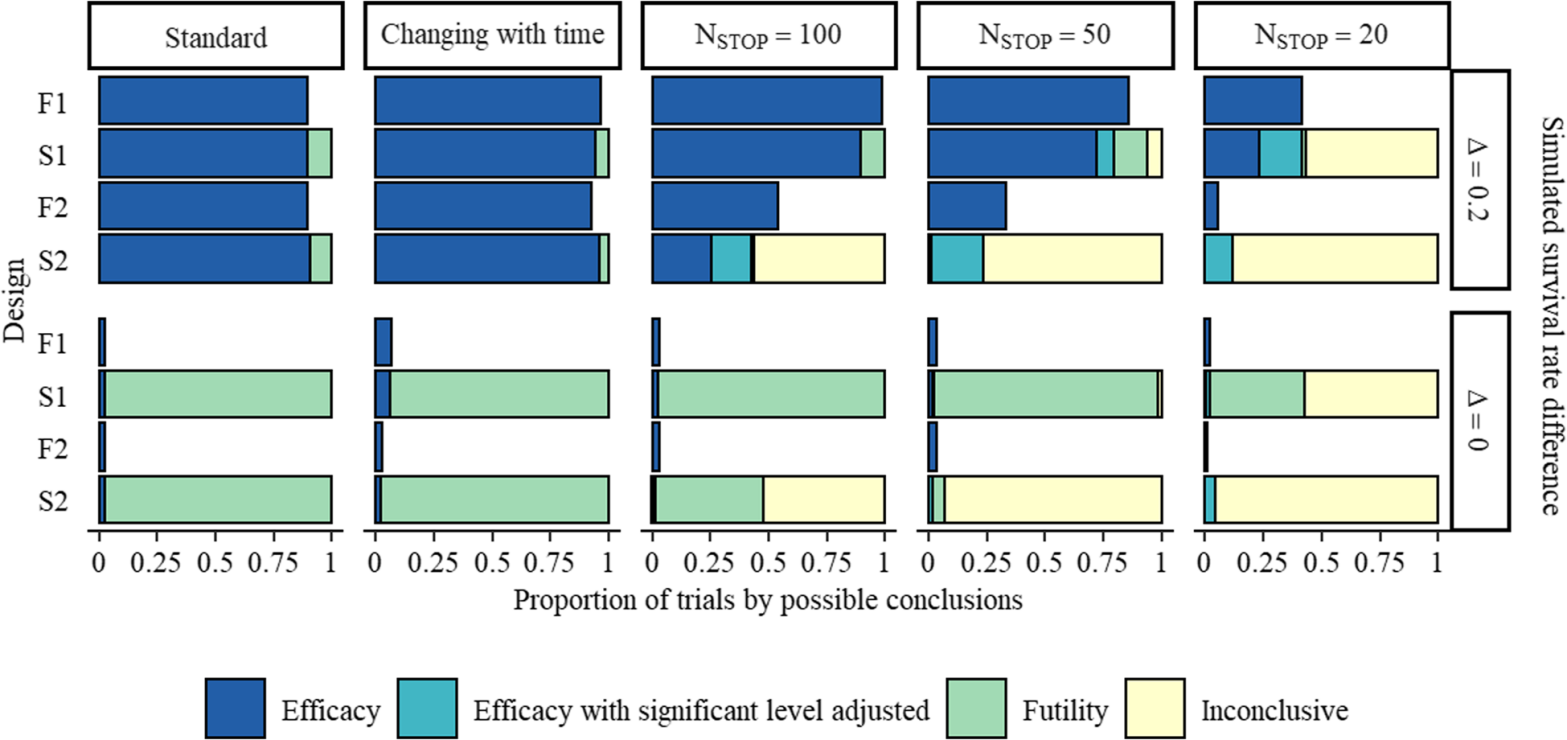
Proportion of significant tests with distribution of conclusions for each design and specific case (p_C_ = 0.50). Abbreviations: p_C_ indicates control survival rate; ∆: simulated survival rate difference; F1: fixed single-arm design; S1: group-sequential single-arm design; F2: fixed two-arm design; S2: group-sequential two-arm design; “Standard”: specific case with a fixed control survival rate; “Changing with time”: specific case with an increase of the control survival rate over time; “Stopping of recruitment”: specific case with a fixed control survival rate and an early stop of the trial due to an outbreak end. For group-sequential trials, the total of trials demonstrating an efficacy of the experimental treatment was defined by the sum of trials with a significant test and trials with an adjusted *p*-value inferior to 0.025. The p-value was adjusted for underrunning by the method proposed by Whitehead [18]

From a methodological standpoint, concern must be taken when conducting a group-sequential trial. Indeed, for “stopping of recruitment” case presenting a premature termination due to an epidemic end, the adjustment for underrunning had an important impact on the results. The number of simulated trials showing a significant efficacy increased after adjusting for underrunning: the adjusted *p*-value led to conclude more often to efficacy of treatment (Fig. 3). Therefore, in the case of an epidemic end, where the cumulative information for group-sequential trials is too low due to the impossibility of including patients, it is necessary to consider the adjusted *p*-value to limit the loss of information and to increase the probability to conclude (for futility or efficacy).

One limitation of our study is that our recommendation on using single-arm trial if it starts after the epidemic peak is based on the assumption made on the pre-trial historical survival rate used to design the trial. Indeed, we noted that results are substantially biased when this value is incorrect and the treatment is harmful. Double-blind randomised controlled trial is the best method to use to avoid those erroneous conclusions. However, conducting a randomised controlled trial is not always feasible. In those cases, single-arm trials should be used when there is relatively strong evidence behind the pre-trial historical survival rate used. Another limitation is that our analyses are based on the widely used 2.5% threshold for the type-I-error, for a one-sided test. However, in the context of viral haemorrhagic fever, where mortality rates are high and few effective therapies are available, a 2.5% threshold may not be appropriate and may even raise ethical questions. This threshold has previously been challenged, and recent studies, especially in oncology, explored new methods (Bayesian Decision Analysis) to choose suitable thresholds that minimize the overall expected harm to patients within clinical trials and future patients especially for the deadliest diseases [20]. In this clinical trial simulation study, we decided to set the type-I-error at 2.5% for a one-sided test. By increasing the type-I-error, it is expected that the number of subjects included will decrease. However, the choice of this threshold is questionable. In particular, it could depend on the disease being studied, the potential outbreak context, the mortality rate, as well as the prevalence (e.g. rare disease).

An extension of this work would be to consider the case of multi-arm clinical trials. In the context of an outbreak, several treatments could be candidates. This was observed for Ebola virus, where various treatments were tested during the same outbreak period [6–8, 12, 13]. Each treatment was evaluated by one clinical trial and associated with a specific research institute and country. Multi-arm trial has the advantage of reducing the number of subjects to be included: indeed, a single control arm is needed against different experimental arms without loss of power. Launching several two-arm trials increases the number of control arms and the global sample size to find the right treatment [21, 22]. It also raises difficulties about acceptability for the participants and lead to a large loss of time due to competing clinical trials. The gain in the number of subjects is an important point in the context of an outbreak where the number of cases decreases following its peak and the choice of treatment to be tested is uncertain. Moreover, group-sequential trials allow to minimise the expected number of subjects included regardless of the number of arms in the trial design. The same considerations explored in this simulation study for two-arm trials would apply to multi-arm trials; except for the case of group-sequential multi-arm design, where after premature termination of treatment arms for futility, more patients would have the possibility to receive the remaining treatment. Comparing the performance of a multi-arm group-sequential trial to a fixed trial at the end of an outbreak would be interesting, but would require to explore a multitude of scenarios, such as the efficacy or futility of each treatment.

In this simulation study, we decided to evaluate a randomization 1:1 for two-arm trial designs. An unequal allocation ratio in favour of the experimental treatment arm would have increased the probability of treatment exposure of the included patient. At first sight, this strategy is beneficial, especially at the end of an outbreak when the number of cases and the number of inclusions is decreasing substantially. However, the loss of power due to this mode of randomization is not negligible: despite an increase in the proportion of patients receiving treatment, this design would therefore require larger sample sizes to achieve the same level of statistical power [23].

Furthermore, this point questions the choice of designs for group-sequential rather than adaptive trials. This type of trial is based on the same principles as group-sequential trials: to adapt the trial based on data accumulated during the study. For example, in a group-sequential trial, a treatment arm will be stopped for futility. With an adaptive trial, it will be possible to re-evaluate during the study the number of subjects needed and change the probability of assigning a treatment (as in the case of unequal allocation ratios).

In practice, adaptive trials are mainly used for the evaluation of treatment doses. They allow, like multi-stage multi-arm trials (MAMS), the combination of phase II and phase III trials. Our simulation study focuses on the performance of phase III clinical trials. Further work could be conducted on adaptive methodologies and MAMS. New questions arise concerning the efficiency of these methods compared with phase II and III clinical trials, with the aim of accelerating the therapeutic evaluation process, using both Bayesian and frequentist approaches [15, 24]. In the case of a VHF outbreak, such as Ebola virus, one MAMS phase II – phase III trial was conducted with a multicentre, multi-outbreak, randomized controlled trial design [25]. In addition, by focusing on adaptive phase III clinical trials, the reasoning would no longer be short-term (a single outbreak wave) but medium and even long-term with withdrawal and/or addition of treatment arms according to the evolution of knowledge about the disease studied, eligible treatments, and marketing authorisations specific to each country. Indeed, an adaptive trial, a MAMS trial, or a classic multi-arm trial seem to be possible over several outbreak waves (multi-outbreak).

Following this work, we wish to create a more complete Shiny App were users could enter other values than that presently available in the drop-down menus.

## Conclusion

In conclusion, the choice of the clinical trial design to be conducted depends on the timeline of the outbreak of a viral haemorrhagic fever. At the beginning of the outbreak, group-sequential two-arm trials should be preferred, as the number of infected cases will increase until the epidemic peak allowing to conduct a strong randomised controlled trial. Moreover, a group-sequential design will allow an early termination of the trial in cases of harmful experimental treatment. The stopping for futility would be faster than the stopping for efficacy, which is an important aspect during first period of an outbreak, usually corresponding to a treatment screening phase.

Regarding trials beginning after the epidemic peak, fixed single-arm design should be performed, as the number of cases decreases after the peak of the outbreak, reducing the number of patients that could be included. However, this assumes that a high level of confidence can be put on the pre-trial historical survival rate used in the single-arm trial.

## Supplementary Information

**Additional file 1.** Description of the assessed clinical trial designs.

## Data Availability

The datasets used and/or analysed during the current study are available from the corresponding author on reasonable request. The R codes for simulations are available in GitHub: https://github.com/PMN-BCH/simu-VHF. All results are available were in the online Shiny App: https://pmn-bch.shinyapps.io/simu-vhf/.

## Abbreviations

F1: Fixed single-arm design
F2: Fixed two-arm design
MSA: Multi-stage approach
NSN: Numbers of subject needed
p_C_: Control survival rate, the proportion of patient who survived in the control arm
p_E_: Experimental survival rate, the proportion of patient who survived in the experimental arm
p_H_: Pre-trial historical survival rate
S1: Group-sequential single-arm design
S2: Group-sequential two-arm design
