# Supplementary material for: Evaluation by simulation of clinical trial designs for evaluation of treatment during a viral haemorrhagic fever outbreak
Source: BMC Med Res Methodol. 2021 May 6;21:98. doi: 10.1186/s12874-021-01287-w (PMC8099711; doi:10.1186/s12874-021-01287-w)
Supplement: Supplementary file 1 — Additional file 1. Description of the assessed clinical trial designs. [file 12874_2021_1287_MOESM1_ESM.docx]

**Additional file 1**

Additional file 1. Description of the assessed clinical trial designs.

|  | **Single-arm**   - **Historical comparison** | **Two-arm**   - **Randomization 1:1** | **Statistical analyses** |
| --- | --- | --- | --- |
| **Fixed design** | F1  H_0_: $\text{p}_{\text{E}}\text{ }\text{=}\text{ }\text{p}_{\text{H}}\text{ }$*vs*.  H_1_: $\text{p}_{\text{E}}\text{ }\text{>}\text{ }\text{p}_{\text{H}}$ | F2  H_0_: $\text{p}_{\text{E}}\text{ }\text{=}{\text{ }\text{p}}_{\text{C}}\text{ }$*vs*.  H_1_: $\text{p}_{\text{E}}\text{ }\text{>}{\text{ }\text{p}}_{\text{C}}$ | Unilateral Z tests  If p-value < 0.025, significant test |
| **Sequential design** | S1  H_0_: Θ = 0 *vs.* H_1_: Θ > 0,  with Θ$\text{=}\text{ }\text{log}\left[ \frac{\text{p}_{\text{E}}\text{(1-}\text{p}_{\text{H}}\text{)}}{\text{p}_{\text{H}}\text{(1-}\text{p}_{\text{E}}\text{)}} \right]$ | S2  H_0_: Θ = 0 *vs*. H_1_: Θ > 0,  with Θ$\text{=}\text{ }\text{log}\left[ \frac{\text{p}_{\text{E}}\text{(1-}\text{p}_{\text{C}}\text{)}}{\text{p}_{\text{C}}\text{(1-}\text{p}_{\text{E}}\text{)}} \right]$ | - Whitehead triangular tests - Stopping boundaries defined with α = 0.025 - Significant test if the test statistic was superior to the upper stopping boundary - Number of interim analyses defined a priori: every 20 patients included |

Abbreviations: $\text{p}_{\text{C}}$indicates control survival rate; p_H_: pre-trial historical survival rate; $\text{p}_{\text{E}}$: experimental survival rate; F1: fixed single-arm design; S1: group-sequential single-arm design; F2: fixed double-arm design; S2: group-sequential double-arm design.
